# Uncertainty Quantification of Microstructure—Governed Properties of Polysilicon MEMS

**DOI:** 10.3390/mi8080248

**Published:** 2017-08-12

**Authors:** Ramin Mirzazadeh, Stefano Mariani

**Affiliations:** Dipartimento di Ingegneria Civile e Ambientale, Politecnico di Milano, Piazza L. da Vinci 32, 20133 Milano, Italy; ramin.mirzazadeh@polimi.it

**Keywords:** microelectromechanical systems (MEMS), polysilicon, coupled electromechanical analysis, stochastic effects, parameter identification

## Abstract

In this paper, we investigate the stochastic effects of the microstructure of polysilicon films on the overall response of microelectromechanical systems (MEMS). A device for on-chip testing has been purposely designed so as to maximize, in compliance with the production process, its sensitivity to fluctuations of the microstructural properties; as a side effect, its sensitivity to geometrical imperfections linked to the etching process has also been enhanced. A reduced-order, coupled electromechanical model of the device is developed and an identification procedure, based on a genetic algorithm, is finally adopted to tune the parameters ruling microstructural and geometrical uncertainties. Besides an initial geometrical imperfection that can be considered specimen-dependent due to its scattering, the proposed procedure has allowed identifying an average value of the effective polysilicon Young’s modulus amounting to 140 GPa, and of the over-etch depth with respect to the target geometry layout amounting to O=−0.09 μm. The procedure has been therefore shown to be able to assess how the studied stochastic effects are linked to the scattering of the measured input–output transfer function of the device under standard working conditions. With a continuous trend in miniaturization induced by the mass production of MEMS, this study can provide information on how to handle the foreseen growth of such scattering.

## 1. Introduction

The advances in the field of integrated circuits and semiconductors had a direct impact on the emergence of a wide range of micro-sized devices, which we know today as microelectromechanical systems, or MEMS [1,2]. Combining electronic and structural components at the microscale, MEMS have been outperforming the conventional technologies in a variety of engineering applications like e.g., accelerometers, magnetometers, scanners, pressure sensors and gyroscopes [3,4].

Due to the continuous growth in the industrial demand for MEMS applications, novel devices are designed to feature ever smaller dimensions, with characteristic sizes of some structural components on the order of 1 μm. The success of these new MEMS critically hinges on their reliability and the predictability of their performances. However, the miniaturization pathway may increase issues related to the mechanical and/or geometrical properties of the fabricated devices [5]. This has forced a focus on assessing the effect of the underlying uncertainty sources at the microscale on the overall performance of the devices (see, e.g., [6]).

The uncertainties in the mechanical properties of the structural parts of MEMS, primarily of the movable structures in case of inertial devices, can stem either from the specific fabrication process adopted [7], or from the fact that their relevant dimensions and the characteristic lengths associated with material heterogeneities (like, e.g., the grain size) become comparable. While the effects of the first cause can be partially reduced by improving the fabrication process, those of the second one are intrinsically linked to the stochastic nature of the material microstructure. All such effects are experimentally testified by the scattering in the measured properties of the devices, see e.g., [8,9,10,11].

The mentioned variations of the geometrical properties of structures obtained through patterning and etching of silicon films are often encountered. For instance, in [12], a variation of around 10% in the dimensions of the movable structure of a polysilicon MEMS was observed; in [1], a 0.2 μm variation was also observed over a 2 μm thick structural component. In [13], capacitive gaps (for electrostatic actuation/sensing) varying between 5.3 and 5.6 μm were reported, against a target value of 5 μm, for a single-crystal MEMS gyroscope. Such variations can obviously differ, depending on the specific production process adopted. With a focus on the mechanical reliability of inertial MEMS whose movable structures are made of polysilicon films, in this paper, we assess the link between microstructural uncertainties due to film morphology and geometry on one side, and the overall performance of the devices on the other side. For devices featuring single-crystalline movable structures, the uncertainties linked to the over-etch depth still play a role in possibly setting reliability and performance issues.

The present work relies upon an on-chip MEMS testing device proposed in [14,15], fabricated using the ThELMA (Thick Epipoly Layer for Microactuators and Accelerometers) surface micromachining process developed by STMicroelectronics (Geneva, Switzerland) (see, e.g., [7,16]), and designed to purposely enhance the sensitivity of the device response to microstructure-governed uncertainties. The scattered response of the device is numerically interpreted through a coupled electromechanical, reduced-order analytical model of the device itself. A genetic algorithm (GA) is then adopted to quantitatively estimate the uncertainty levels linked to the film mechanical properties, to the etch variation or over-etch depth [17,18] and to a possible initial offset or geometrical imperfection of the structure, leading to asymmetry in the response. As reported in [15], a Bayesian inference method based on particle filtering may show some instabilities close to the pull-in of the devices, in terms of estimated values of the aforementioned mechanical and over-etch properties tending to diverge from formerly attained quasi steady-state solutions. To avoid such effects and correctly trusting all the measurements acquired during the experimental campaign, a batch approach like GA is preferred here. GAs have been already adopted for the robust optimization of MEMS structures (see, e.g., [19,20,21]), but, to the best of our knowledge, not for parameter identification purposes.

The remainder of this work is organized as follows. In Section 2, a description of the on-chip testing device is reported, and the details of an analytical model that accounts for the deformation of a microstructured thin beam are provided, in order to assess the effects of beam stiffness, geometry and initial offset on the input–output relation of the device. Section 3 deals with the adopted GA to estimate the values of the parameters governing the beam response to loading and the initial imperfection, in order to optimally match the experimental data collected by testing a set of nominally identical devices. A statistical analysis of the results is next reported in Section 4. Finally, some concluding remarks and suggestions for future investigations and further improvements of the proposed model/procedure are collected in Section 5.

## 2. On-Chip Testing Device: Experiments and Modeling

**Figure 1:**
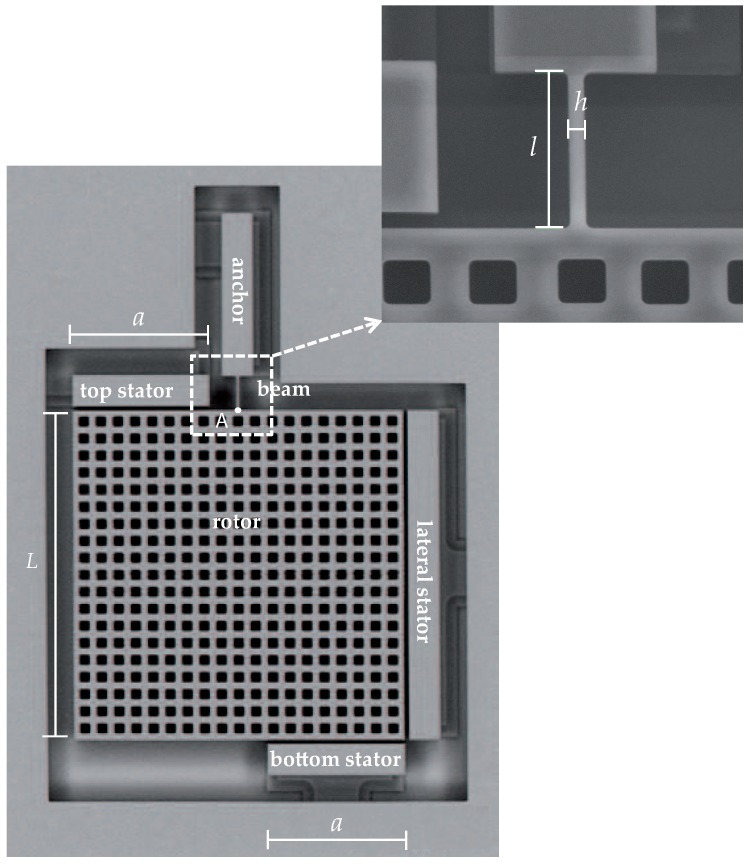
SEM picture of the on-chip testing device, with a close-up of the polysilicon beam sample.

The mentioned ThELMA process allows for producing thick polysilicon films, grown on top of the die substrate, with a columnar microstructure. The micromechanical characterization of the film has been carried out in this work with the on-chip testing device proposed in [14,15] and shown in Figure 1. Although devices with a different geometry have been patterned on a single die and capped through a glass frit-based low temperature wafer-to-wafer bonding technique inside a single cavity (see [22,23]), in what follows, we will focus on the structure featuring the dimensions reported in Table 1.

The tested microstructured specimen is represented by the thin beam at the top side of the massive plate. This beam provides the only link between the suspended plate and the anchor to the substrate: under the types of electrostatic actuation described in what follows, the plate is displayed as a rigid body and the only deformed structural part of the device is the beam itself. The featured out-of-plane thickness *w* = 22 μm of the beam, in comparison with its in-plane width h=2 μm, allows for assuming that plane-strain conditions rule the problem at the beam level. The production process guarantees that the lateral surfaces of the movable parts of the device, and of the surrounding stators adopted for actuation and sensing are all flat, with sidewall scallops [24] of negligible effect on the measured response.

**Figure 2:**
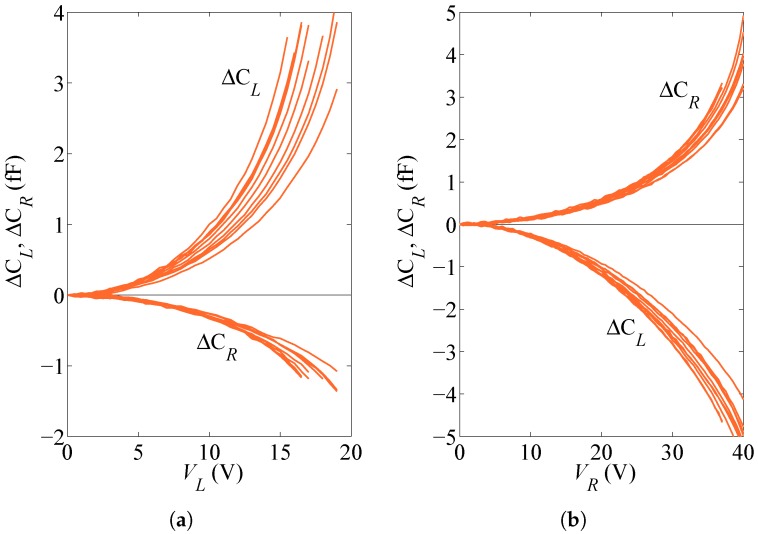
Experimentally measured capacitance change values $\Delta C_{L}$ and $\Delta C_{R}$ under (**a**) lateral actuation through $V_{L}$, and (**b**) rotational actuation through $V_{R}$.

Testing of each polysilicon sample is obtained with a bias voltage applied either as $V_{L}$ to the lateral stator or as $V_{R}$ to the top and bottom stators; the rotor, the beam and the anchor are instead electrically grounded. Through $V_{L}$, the electrostatic actuation provides a lateral force (horizontal in Figure 1), which induces both a shear and a bending of the beam; through $V_{R}$, the actuation gives rise instead to an in-plane torque and so to a pure bending of the beam with no axial and shear forces. While the structural system can be assumed to behave linearly due to the small displacement and rotation experienced by the plate up to pull-in instability [25], the electrostatic actuation at the parallel (or slightly tilted, see the discussion below related to possible initial imperfections) surfaces of the capacitors provides actuation forces that are nonlinear functions of the gap in the narrow electrical domains; these forces are effectively modified by the specimen deformation. Moreover, the specimen response is measured via the capacitance changes (with respect to the relevant values at zero actuation) at the same capacitors, say $\Delta C_{L}$ for the lateral capacitor and $\Delta C_{R}$ for the two rotational capacitors; the capacitance changes are again nonlinear functions of the beam deformation. Overall, the output capacitance change vs. input voltage relationships turn out to be nonlinear; actuation can be progressively increased up to when the attraction forces at capacitors become too large to be counteracted by the stiffness of the beam, and pull-in instability occurs as marked by the capacitance value growing to infinity. The experimental results obtained for a set of ten nominally identical devices, represented in terms of $\Delta C_{L}$ and $\Delta C_{R}$ as functions of an applied voltage $V_{L}$ or $V_{R}$, are shown in Figure 2.

According to the qualitative description of the device behavior provided here above, it emerges that discerning the effects of material uncertainties linked to the polysilicon morphology in the beam and of geometrical uncertainties linked to over-etch depth, on the measured response of the device, is a nontrivial task. Hence, an identification procedure, based on a GA, is proposed in Section 3 to assess the mentioned uncertainties from the scattering in the responses of Figure 2, all related to the nominally identical specimens.

Moving now to the formulation of the analytical model of the device, accounting for the beam slenderness measured through its length/in-plane width ratio, the Timoshenko theory for thin or moderately thick beams is adopted. For a homogeneous micro-cantilever beam, the relationship between the load components at its tip (point A in Figure 1), in terms of shear force *F* and in-plane torque *M*, and the induced deflection *u* and rotation θ then reads:

(1) $$\left\{\begin{array}{c} (1+\psi_{T})F\\ M \end{array}\right\}=\frac{EI}{l^{3}}\left[\begin{array}{cc} 12 & -6l\\ -6l & 4l^{2} \end{array}\right]\left\{\begin{array}{c} u\\ \theta \end{array}\right\}$$

where (see also Table 1 and Figure 1): *l*, *h* and *w* are, respectively, the length, in-plane width and out-of-plane thickness of the beam; $I=wh^{3}/12$ is the in-plane moment of inertia of the beam cross-section; *E* is the effective Young’s modulus of the beam, accounting for the elastic orthotropy of each FCC silicon grain [26] and for the polycrystalline morphology [27,28]; EI represents the flexural rigidity of the cantilever; $\psi_{T}$ is a dimensionless factor that accounts for the shear deformations in the beam and is a function of the so-called shear correction factor (see, e.g., [29] for anisotropic beams). Due to the already mentioned small gap between the conductors (amounting to $g_{0}$ in the initial, ideal offset-free configuration), pull-in happens for this device when the assumption of a linearized kinematics is still valid.

The loading terms *F* and *M* in Equation (1) are provided by the electrostatic actuation. As already discussed in [15], due to the small rotation of the rotor for any admissible value of $V_{L}$ and $V_{R}$, along the faces of the conductors, the local solution for parallel plate capacitors can be adopted to provide the densities (per unit area) of the attraction force as:

(2) $$f=\frac{\epsilon V^{2}}{2g^{2}}$$

and of the capacitance as:

(3) $$c=\frac{\epsilon }{g}$$

where: $\epsilon =\epsilon_{r}\epsilon_{0}$; $\epsilon_{r}$ is the relative permittivity of the medium, and $\epsilon_{0}$ is the dielectric constant in the free space; *g* is the local gap in the electrical domain; and *V* is the applied voltage (either $V_{L}$ or $V_{R}$, depending on the actuation type). When the beam deforms, the gaps $g_{L}$ and $g_{R}$, once again across the electrical domains for the lateral and the top-bottom capacitors, can be linked to the displacement *u* and rotation θ at the cantilever tip via:

(4) $$\begin{array}{cc} g_{L}= & g_{0}-u-x_{2}\sin\theta \\ g_{R}= & g_{0}+x_{1}\sin\theta \end{array}$$

where, according to what was depicted in Figure 1, it is assumed that *u* is positive if pointing to the right, and θ is positive if counterclockwise.

Due to the high ratio between the in-plane length *L* (*a*) of the surface of the lateral (top-bottom) stator and the reference gap $g_{0}$, fringe effects are disregarded. Hence, upon integration over the capacitor surfaces, the electrostatic actuation turns out to provide, in the lateral case:

(5) $$\begin{array}{cc} F_{L}= & \frac{\epsilon wV_{L}^{2}L}{2\left(g_{0}-u\right)\left(g_{0}-u-L\sin\theta \right)}\\ M_{L}= & \frac{\epsilon wV_{L}^{2}}{2\sin^{2}\theta }\left(\frac{L\sin\theta }{g_{0}-u-L\sin\theta }+\log\left(\frac{g_{0}-u-L\sin\theta }{g_{0}-u}\right)\right) \end{array}$$

and in the rotational case:

(6) $$\begin{array}{cc} F_{R}= & 0\\ M_{R}= & \frac{\epsilon wV_{R}^{2}}{\sin^{2}\theta }\left(\log\left(\frac{g_{0}+\left(\frac{L}{2}-a\right)\sin\theta }{g_{0}+\frac{L}{2}\sin\theta }\right)+\frac{ag_{0}\sin\theta }{\left(g_{0}+\left(\frac{L}{2}-a\right)\sin\theta \right)\left(g_{0}+\frac{L}{2}\sin\theta \right)}\right) \end{array}$$

As for sensing, the values of capacitance between the facing plates in the two displaced configurations respectively read:

(7) $$\begin{array}{cc} C_{L}= & \frac{\epsilon w}{\sin\theta }\log\left(\frac{g_{0}-u}{g_{0}-u-L\sin\theta }\right) \end{array}$$

(8) $$\begin{array}{cc} C_{R}= & \frac{2\epsilon w}{\sin\theta }\log\left(\frac{g_{0}+\frac{L}{2}\sin\theta }{g_{0}+\left(\frac{L}{2}-a\right)\sin\theta }\right) \end{array}$$

As already pointed out, the device output is actually measured through the capacitance changes, i.e., the variations $\Delta C_{L}$ and $\Delta C_{R}$ with respect to the values $C_{L0}$ and $C_{R0}$ in the initial (either affected by imperfection or offset-free) configuration. Formulae reported here above are similar to those in [15]; the differences between the two sets of equations are only due to a different representation of the logarithmic terms.

The model discussed so far is purely deterministic. To account for uncertainties at the micro-scale, terms related to the variability of the response of the device under fluctuations of stiffness and geometry of the structure must be considered.

**Figure 3:**
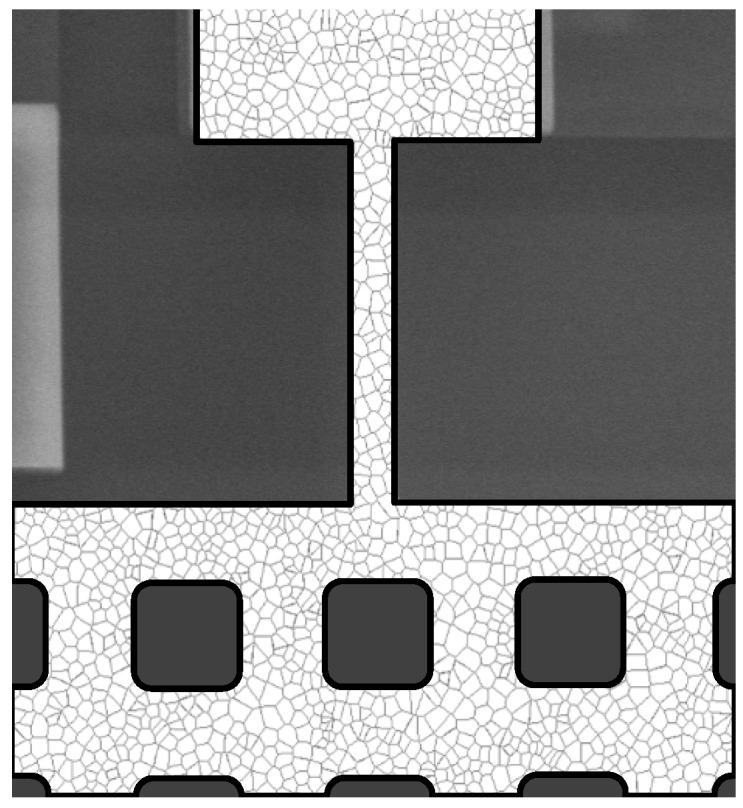
Close-up of an example of (digital) polycrystalline morphology of the movable structure.

As far as the mechanical properties of the polysilicon film are concerned, the parameters coming into play in the model are the effective elastic moduli *E* and *G* of the beam, the latter being implicitly accounted for through the coefficient $\psi_{T}$ in Equation (1). To provide insights into the effect of the polycrystalline morphology on the effective stiffness of the beam, the results of homogenization on a statistical volume element can be resorted (see, e.g., [27,30]). By increasing the size of the polycrystalline sample, bounds on or estimates of the elastic moduli all converge to the asymptotic values in accordance with e.g., [31]; for small samples, featuring a few grains only across the entire width of the beam (see Figure 3), results get much scattered. The relevant scattering can then be assessed by continuously varying the beam microstructure in a Monte Carlo analysis (see also [32]). Alternatively, the effective elastic moduli of the beam can be identified by handling the experimental plots of Figure 2 for each single specimen, finding the optimal values of the moduli matching at best the data, and then assessing the variability of these values within the set of tested devices.

As far as the geometrical uncertainties are concerned instead, two different sources can be considered. First of all, the initial structural configuration can be characterized by an offset, or geometrical imperfection induced by the processes of deposition and release of the polysilicon film. Within the proposed framework, such imperfection is parametrized by the displacement $u_{0}$ and the rotation $\theta_{0}$ at the cantilever tip under no actuation. Such values affect the gap across the electrical domains, and so modify both actuation and sensing. In addition to that, the etching process necessary to release the movable structure from the substrate also induces a so-called over-etch, which can be defined as a defect in the in-plane geometry due to a fluctuation of, for example, temperature and etchant concentration (see [15,18,33]). If *O* identifies the over-etch depth, assumed to be isotropic in the plane of motion of the structure, in the ideal configuration, the main geometric features of the device are modified according to:

(9) $$\begin{array}{cc} h^{★}= & h-2O\\ l^{★}= & l+2O\\ g_{0}^{★}= & g_{0}+2O \end{array}$$

The deviation *O* from the target geometry, and so the sample-dependent lengths $h^{★}$, $l^{★}$ and $g_{0}^{★}$, can be estimated too by matching the experimental data.

To account for all of the above-mentioned sources of uncertainties at the microscale, the values of *E*, *G*, *O*, $u_{0}$ and $\theta_{0}$ should be identified for each single specimen by minimizing a measure of the discrepancy between the applied voltage vs. capacitance change responses depicted in Figure 2, and the relevant model outcomes. To avoid the typical issues ruled by curse of dimensionality in identification problems, see e.g., [34], that can be even amplified by the nonlinear equations governing actuation and sensing, the set of parameters to be tuned has to be analyzed first.

Concerning the effective elastic moduli, while *E* has a major role in Equation (1), *G* affects the solution only by modifying the beam compliance to allow for shear deformations; due to the beam slenderness and accounting for the results of [27], where it was shown that in-plane elastic isotropy can be attained for rather small volume elements of the polysilicon film, *G* is set in what follows according to $G=\frac{E}{2(1+\nu )}$, where Poisson’s ratio is assumed to be ν=0.175 (see again [27]). The microstructure-dependent value of *G* is therefore directly correlated to the variability of the effective Young’s modulus *E*, which becomes the only elastic parameter handled by the identification procedure.

**Figure 4:**
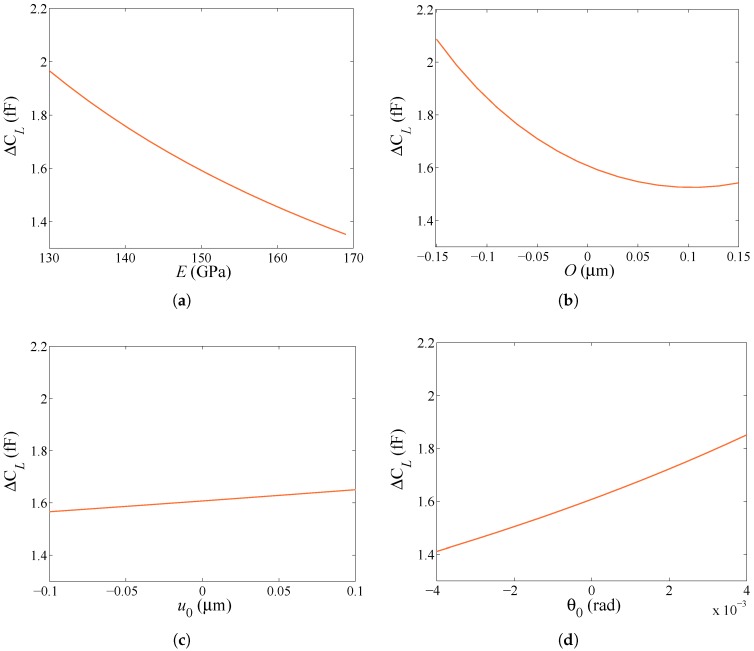
Lateral actuation through $V_{L}=15$ V, sensitivity of the capacitance change $\Delta C_{L}$ to: (**a**) *E*; (**b**) *O*; (**c**) $u_{0}$; (**d**) $\theta_{0}$.

**Figure 5:**
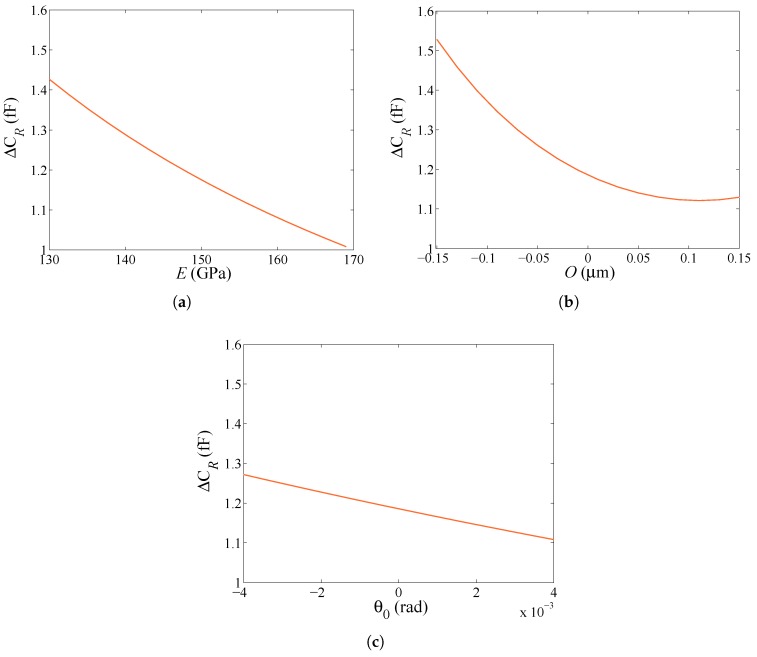
Rotational actuation through $V_{R}=30$ V, sensitivity of the capacitance change $\Delta C_{R}$ to: (**a**) *E*; (**b**) *O*; (**c**) $\theta_{0}$.

To assess now the effect of parameters *E*, *O*, $u_{0}$ and $\theta_{0}$ on the measurables, the relevant sensitivities of the capacitance change $\Delta C_{L}$ under an actuation of $V_{L}=15$ V, and of the capacitance change $\Delta C_{R}$ under an actuation of $V_{R}=30$ V are respectively reported in Figure 4 and Figure 5. In Figure 4, it is shown that the sensitivity (as quantified by the slope of the plots) to $u_{0}$ is rather limited in the considered intervals, which have been set as follows. *E* is assumed to vary in the range E∈[$E_{min}=130$ GPa, $E_{max}=169$ GPa], see [26], bounded by the values relevant to single-crystalline silicon for rotations in the plane perpendicular to one crystal lattice orientation. In accordance with the production process, *O* and $u_{0}$ are respectively assumed to vary in the ranges O∈[$O_{min}=-0.15$ μm, $O_{max}=0.15$ μm] and $u_{0}\in$[$u_{0,min}=-0.1$ μm, $u_{0,max}=0.1$ μm]. Finally, the range of variation for $\theta_{0}$ is set by considering the pull-in instability, so that $\theta_{0}\in$[$\theta_{0,min}=-4$ mrad, $\theta_{0,max}=4$ mrad]. In Figure 5, the sensitivity to $u_{0}$ is not reported, as it is assumed null in the case of rotational actuation. In fact, under a lateral actuation pull-in occurs for $u_{0}^{\mathrm{pull-in}}=\frac{g_{0}}{3}=0.67$ μm; under rotational actuation, this value of $u_{0}^{\mathrm{pull-in}}$ would induce a variation of the surface of the facing conductors at top/bottom stators, and so of the in-plane electrostatic-induced torque $M_{R}$ (the only non zero actuation term in this case) amounting to $\frac{0.67}{83}\approx 0.8\%$. For the considered range of $u_{0}$ reported above here, the variation would be even smaller and surely masked in the experiments by the noise level shown in Figure 2. According to this reasoning and to what reported in Figure 4c, the identification problem can then be focused on the estimation of *E*, *O* and $\theta_{0}$ only. Remarkably, the sensitivity analysis cannot be adopted alone for identification purposes, as the effects of *E* and *O* are shown to be rather similar: in fact, a more compliant beam, due to either a less-stiff material or a thinner cross-section, provides higher values of $\Delta C_{L}$ and $\Delta C_{R}$, independently of the type or actuation. Vice-versa, positive values of $\theta_{0}$ induce a softening (and higher values of the capacitance change) under a lateral actuation and stiffening under a rotational actuation. To properly distinguish each effect, a GA is then adopted to govern the identification procedure.

## 3. Identification Procedure: Genetic Algorithm

GAs are heuristic computational algorithms, mainly based on the evolutionary ideas of genetics and natural selection (see [35,36,37,38]).

Through simplifications of the evolutionary theory, GAs can be considered as stochastic optimization engines that handle a population of candidate solutions (also known as individuals or phenotypes), and employ heuristics such as selection, crossover and mutation to evolve such individuals over sequential generations. The basic steps of the method can be summarized as follows:

1. Generate a random initial population composed of $n_{P}$ candidate solutions;
2. Evaluate the fitness for each member of the current population through a purposely defined objective function;
3. Select the so-called parents, i.e., members better fitting the object, for reproduction purposes;
4. Pass a number of so-called elite parents to the next generation population;
5. Generate offspring by combining the elements (or chromosomes) of two parents, and by applying variations (based on any specific probability distribution) to a parent or to the newly generated offspring. The first operator is called crossover, whereas the second one is termed mutation;
6. Replace the current population with the newly generated offspring;
7. Perform steps 2 to 6 until a termination condition is met, in terms of either maximum number of generations or minimum change in the value of the best fitness among subsequent generations.

The initial distribution of individuals should be in accordance with the a priori knowledge (also called expert guess in other contexts) of the most probable values or distributions of the parameters governing the problem/model. In the absence of such information, a general practice is to use a random distribution of the population over the search space, so as to increase the chance of accurately locating the optimal solution.

As reported here above concerning the offspring generation, besides handling the elite members, crossover and mutation are both used. The former operator enables the algorithm to combine the elements of the best parents to generate possibly superior offspring; the latter instead prevents the loss of diversity [37] by making random changes in the elements of each individual. GAs differ from one another depending on how operators are tailored to form the next generation. In the current work, 5% of individuals with the best fitness value has been passed to the next generation as the elite. As for the evolution of the other individuals, a uniform crossover with a probability of 50% and a uniform mutation with a probability of 1% have been adopted. Such crossover implies that the elements of the individuals are randomly taken from the parents’ ones.

The population size to explore the parameter space is another important factor to set the effectiveness of the procedure [39,40]. A large population would clearly imply numerous evaluations and thus a high computational cost; on the other hand, such a large population would be advantageous, as it allows a thorough space search and thus a higher chance to find the optimal solution in the case of objective functions characterized by multiple local minima/maxima. In the current work, a population size $n_{P}=5000$ has been managed to estimate the values of the unknown parameters *E*, *O* and $\theta_{0}$ for each tested sample. The procedure has been set to terminate after four successive generations exhibiting no improvement (or a marginal improvement) of the best fitness value.

## 4. Results

Through the use of the GA detailed in Section 3, the values of the free model parameters *E*, *O* and $\theta_{0}$ have been calibrated for each specimen by minimizing a scalar measure of the discrepancy between the experimental and the model responses. For this purpose, the following discrete objective function ϕ has been defined:

(10) $$\phi =\sum_{i=1}^{N}\left(|\Delta C_{L}^{\exp,i}-\Delta C_{L}^{\mathrm{mod},i}|+|\Delta C_{R}^{\exp,i}-\Delta C_{R}^{\mathrm{mod},i}|\right)$$

where: *N* is the number of sampling points, evenly spaced by 0.5 V across the adopted ranges of actuation voltage; for each value of the actuation voltage, $\Delta C_{L}^{\exp,i}$ and $\Delta C_{R}^{\exp,i}$ are the corresponding values of the capacitance changes experimentally measured, whereas $\Delta C_{L}^{\mathrm{mod},i}$ and $\Delta C_{R}^{\mathrm{mod},i}$ are those provided by the model; |⋄| stands for the absolute value of term ⋄. Since the range of values for the capacitance changes are all similar, independently of the actuation type, the two terms related to lateral and rotational sensing are summed without any scaling or weighting factor (see, e.g., [41]). Since, in [15], it was shown that Bayesian parameter estimates have a tendency to diverge from a rather voltage-independent solution when pull-in is approached, the two terms in Equation (10) have also not been downscaled by a factor increasing with the capacitance change. Function (10) thus exploits the sensing modes, and so the possible redundancy in the data, by mixing the two relevant discrepancy terms. In this objective function, while *E* and *O* have a direct effect on the currently measured values of the capacitance at conductors, the rotation angle $\theta_{0}$ describes a distortion of the initial geometry of the device and so affects the values of $C_{L0}$ and $C_{R0}$ at no actuation; since values of $\Delta C_{L}$ and $\Delta C_{R}$ are actually measured and collected from the model, all three of the parameters have an impact on the solution as shown by the sensitivity analysis of Figure 4 and Figure 5.

Although not discussed in details in Section 2, the question as to whether the over-etch depth can be considered isotropic in the plane of motion of the structure is still under debate. To simplify the model or, in other words, to reduce the number of parameters to be identified, it has been assumed that *O* is direction-independent. If this assumption did not allow to describe appropriately the real geometry of the system after the etching phase, the value of the gap $g_{0}^{★}$ (see Equation (9)) would be different across the electrical domains for lateral and rotational actuation/sensing; accordingly, the measured responses would be affected differently by *O*. To partially cope with this additional problem related to the production process of the devices, the objective function ϕ in Equation (10) is obtained by summing the contributions related to lateral and rotational sensing, but not those coming from the different kinds of actuation. Two different optimization problems are then solved for each tested device, depending on whether the lateral or rotational actuation is enforced.

The solutions provided by the GA for the ten tested samples, in terms of actuation-dependent estimated values of *E*, *O* and $\theta_{0}$, are collected in Table 2 together with a statistical analysis providing mean and standard deviation for each parameter. As already discussed in [15], for some specimens, the solution turns out to be independent of the actuation type, within the accuracy featured by the present stochastic frame; for some others, the solution is instead greatly dependent on the actuation type. The difference between the two solutions under either $V_{L}$ or $V_{R}$ can be partially related to the already mentioned anisotropic over-etch depth. However, to specifically address the major difference reported for the values of *E*, anchor compliance [42] should be accounted for. In this regard, according to the data relevant to *E*, we can state that values very close to the lower bound $E_{min}=130$ GPa allowed for polysilicon can be thought of as a weighted average of the bending film stiffness, which affects the solution along the whole length of the beam, and of the anchor stiffness, which is instead a concentrated term related only to the top end cross-section of the beam. This specific topic will be investigated in future works and is not further discussed here, since a proper evaluation of the anchor compliance would require three-dimensional analyses of the anchor region and Monte Carlo simulations to also assess the superimposed effect of the polysilicon morphology.

**Figure 6:**
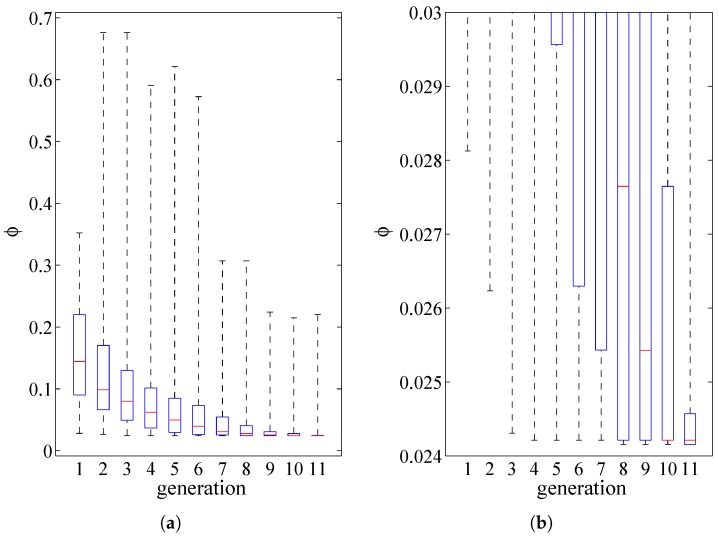
Example of the convergence of the optimal solution provided by the GA at increasing number of generations: (**a**) boxplot showing the spreading of the values of the objective function relevant to all the individuals, and (**b**) close-up near the optimum.

The rather fast convergence of the GA is shown in Figure 6, where boxplots (with no outliers) are adopted to depict the spreading of the values of the objective function over all the individuals, at increasing number of generations. This solution is related to the case of specimen #2 under rotational actuation through $V_{R}$, but similar results have been obtained for all the samples, independently of the actuation type. To better assess the convergence towards the optimal parameter values, besides the plot in Figure 6a that mainly shows the spreading of the values, a close-up is reported in Figure 6b to show how the median (and not only the best values associated to minima) is greatly improved from the eighth generation onward.

**Figure 7:**
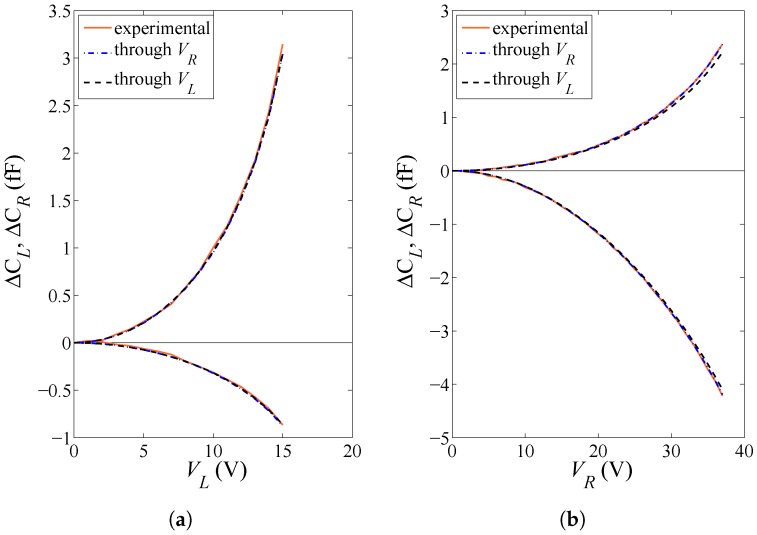
Specimen #9: capacitance change values $\Delta C_{L}$ and $\Delta C_{R}$ under (**a**) lateral actuation through $V_{L}$ and (**b**) rotational actuation through $V_{R}$. Comparison between the experimentally measured response, and the theoretical solution with model parameters tuned by the GA under either lateral or rotational actuation.

**Figure 8:**
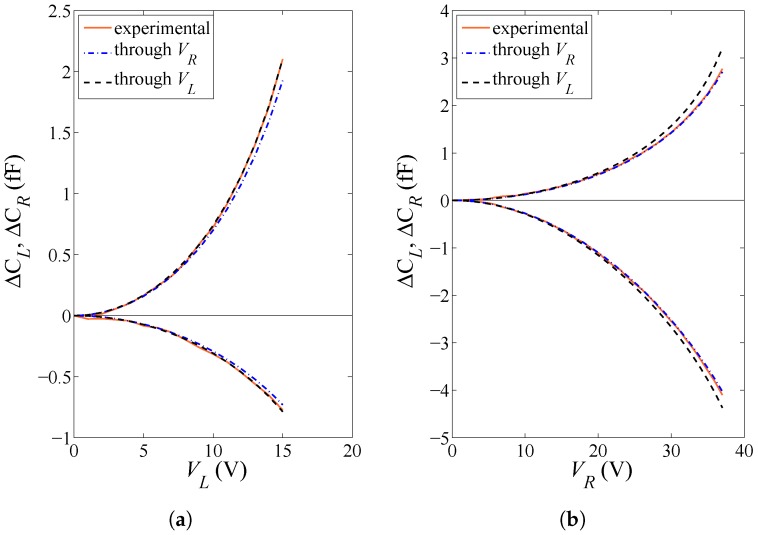
Specimen #4: capacitance change values $\Delta C_{L}$ and $\Delta C_{R}$ under (**a**) lateral actuation through $V_{L}$ and (**b**) rotational actuation through $V_{R}$. Comparison between the experimentally measured response, and the theoretical solution with model parameters tuned by the GA under either lateral or rotational actuation.

By comparing the estimated values of *E* and *O* gathered in Table 2 and the same values estimated through the particle filtering procedure in [15], and by considering as a figure of merit the agreement between the values obtained handling the data related to the two actuation types, it clearly emerges that $\theta_{0}$, being an additional variable of the inverse problem, may improve the effectiveness of the approach, meant as the capability to provide an actuation-independent solution. For a validation of the outcomes of the parameter identification task, a comparison between model and experimental applied voltage vs. capacitance change plots are therefore reported in Figure 7 and Figure 8 for specimens #9 and #4, respectively. The solution corresponding to the former specimen is almost actuation-independent, and so it is expected that the parameter values tuned using either $V_{L}$ or $V_{R}$ can accurately match all the experimental curves. The other way around, the solution corresponding to the latter specimen depends much on the type of actuation, especially concerning *E*; therefore, the model responses obtained through the two different sets of parameters may be somehow different from each other. Overall, in all the cases, the solution provided by the GA allows for perfectly matching the two experimental $\Delta C_{L}$ and $\Delta C_{R}$ curves obtained under the same actuation. Hence, actuation-dependent solutions not perfectly matching all the experimental data have to be ascribed to a partial inaccuracy of the theoretical model to cope with all the uncertainty sources, rather than to a failure of the adopted identification procedure.

**Figure 9:**
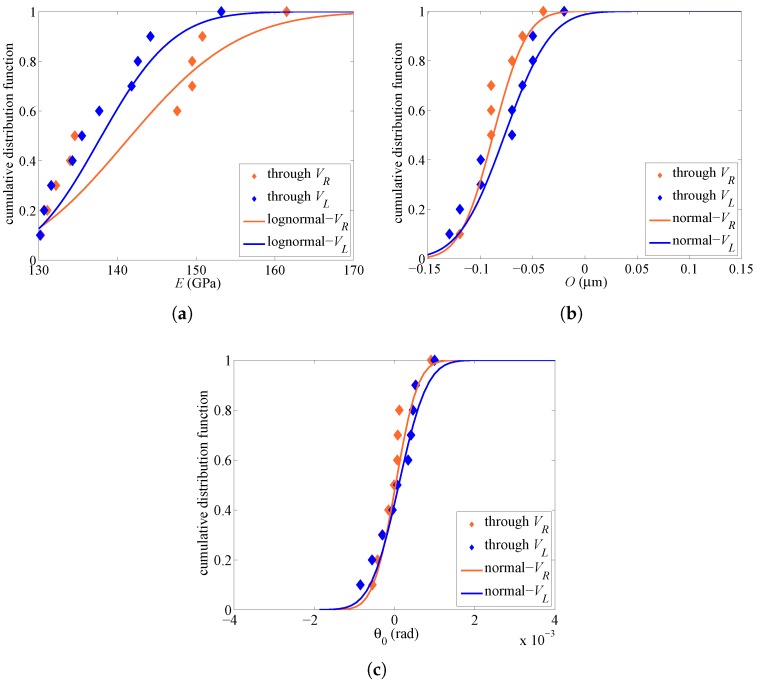
Cumulative distribution functions of model parameters (**a**) *E*, (**b**) *O* and (**c**) $\theta_{0}$ estimated by the GA under either lateral or rotational actuation, and relevant fitting curves.

To finally assess the spreading of the identified solutions for all the tested specimens, and so the possible variability of parameters within a single wafer, in Figure 9, the distribution functions of *E*, *O* and $\theta_{0}$ are gathered. Due to the limited number of samples tested (but keeping in mind that these microscale tests are quite time-consuming to carry out due to the coupled electromechanical physics governing the system response, the extremely small values of the capacitance change to be measured, and the handling of tiny devices in a hopefully clean room), results are reported in terms of cumulative distribution functions. *O* and $\theta_{0}$ values, although not symmetrically distributed around a zero mean, have been fitted using Gaussian or normal distributions; in fact, even with marginal or negligible probability, values far from the relevant medians can still be physically-sound. *E* values have been instead fitted using a lognormal distribution, allowed for to surely (in statistical sense) avoid negative values. Two different fitting functions have been adopted for each parameter, depending on the type of actuation; relevant parameters shaping the fitting distributions are all collected at the bottom of Table 2. From the three graphs, it clearly emerges once more that *O* and $\theta_{0}$ are well estimated, with a rather limited and slightly actuation-dependent scattering. *E* looks instead too scattered across the tested devices, with a remarkable dependence on the actuation type; in statistical terms, a non-negligible probability of values smaller than the lower bound $E_{min}=130$ GPa is obtained. Such unphysical outcomes can be justified, at least partially, by having disregarded the said additional beam compliance at the anchor.

## 5. Conclusions

In this paper, a problem of uncertainty quantification at the microscale has been approached. The analysis has been motivated by the continuous demand for further miniaturization in the MEMS industry. Some reliability and predictability problems have been already encountered when dealing with inertial devices with a characteristic size of some structural members comparable with the internal length-scale of polycrystalline silicon films.

A microsystem has been purposely designed for on-chip testing. To simplify as much as possible the stress state in the tested polysilicon samples, a statically determinate movable structure, consisting of a massive plate (rotor) connected to the substrate through a cantilever beam, has been electrostatically actuated thanks to stators placed around the plate itself. Actuation and sensing have been designed to provide two different types of deformation in the microstructured beam (pure bending under rotational actuation, and coupled bending/shear under lateral actuation) and so to allow collecting redundant experimental data.

Due to the small ratio between the target in-plane width of the beam and the characteristic size of the silicon grains, experimental data (in terms of measured capacitance change vs. applied voltage) have shown a remarkable scattering that cannot be caught by accounting only for the uncertainties related to the effective elastic properties of the polysilicon film. Additional uncertainty sources linked to the geometry of the movable structure have been then considered, in terms of imperfections induced by the patterning/etching phases of the production process and quantified by the so-called over-etch depth and by an initial offset (caused also by possible gradients of the residual stresses). To provide an interpretation of the experimental data and of the scattering induced by all the mentioned uncertainty sources, an analytical (reduced-order) coupled electromechanical model of the device has been provided.

To reduce to a minimum possible issues related to the handling of the experimental data close to pull-in instability, a genetic algorithm has been next adopted to tune the elasticity- and geometry-related free parameters of the model, by matching the response of each single device tested in the experimental phase of this study. The outcomes of this parameter identification procedure have been finally collected altogether, and a statistical dispersion analysis has been provided. The offset has been parametrized by an initial tilting of the plate, which has shown a large scattering quantified through a standard deviation of 0.44 and 0.56 milliradians, respectively, in the case of rotational or lateral actuation. The values of the effective polysilicon Young’s modulus *E* and of the over-etch depth *O*, averaged over all the tested samples, have been instead estimated to amount to: E=142.1 GPa, O=−0.09 μm in the case of rotational actuation; E=138.2 GPa, O=−0.08 μm in the case of lateral actuation. These values and also the relevant standard deviations agree quite well with each other, so they can be considered as physically related to micromechanical features of the film constituting the movable structure of the device, and not induced instead by artifacts due to the actuation mechanisms or to the identification procedure.

While the parameters related to geometrical imperfections have been finally described well via Gaussian probability distributions, the beam effective elasticity has been shown to be too scattered to be described via a physically-sound lognormal distribution. This latter result has been partially related to having neglected a further compliance term at the beam anchor, which is typically negligible at the macroscale but can become of importance at the microscale, in the case of slender beams connected to the substrate via a single anchor region.

To further increase the accuracy of the results, a full-order, coupled electromechanical finite element analysis of the movable structure and of the surrounding fluid will be considered in future investigations. To properly account for the stochastic effects induced by the polysilicon morphology in the beam, a Monte Carlo procedure will be used. To reduce the overall computational burden of the finite element-driven Monte Carlo simulations, model order reduction procedures will be proposed, so as to finally quantify, with a high degree of fidelity, the microstructure- and production process-induced uncertainties in inertial MEMS.

## Figures and Tables

**Table 1:** Dimensions of the on-chip testing device.

Parameter	Value
beam length (*l*)	20 μm
beam width (*h*)	2 μm
out-of-plane beam thickness (*w*)	22 μm
target gap at capacitors ($g_{0}$)	2 μm
top/bottom plate length (*a*)	83 μm
lateral plate length (*L*)	200 μm

**Table 2:** Spacimen- and actuation-dependent values of model parameters *E*, *O* and $\theta_{0}$ estimated by the GA.

	*E* (GPa)		*O* (μm)		$\theta_{0}$ (milliradian)	
Specimen #	through $V_{R}$	through $V_{L}$	through $V_{R}$	through $V_{L}$	through $V_{R}$	through $V_{L}$
1	134.6	131.6	−0.10	−0.05	0.08	0.34
2	147.6	137.7	−0.09	−0.02	−0.15	0.07
3	150.8	153.2	−0.09	−0.13	−0.31	−0.30
4	149.5	130.7	−0.12	−0.07	−0.01	−0.05
5	149.5	141.8	−0.09	−0.10	−0.55	−0.56
6	161.5	144.2	−0.07	−0.07	−0.42	−0.85
7	130.3	134.3	−0.10	−0.10	0.07	0.41
8	134.0	130.2	−0.12	−0.06	0.12	0.46
9	131.1	135.5	−0.04	−0.05	0.91	1.00
10	132.2	142.6	−0.06	−0.12	0.52	0.53
mean	142.1	138.2	−0.09	−0.08	0.03	0.10
standard deviation	10.9	7.3	0.03	0.03	0.44	0.56
